# Synergistic inhibitory activity of Glycyrrhizae Radix and Rubi Fructus extracts on biofilm formation of *Streptococcus mutans*

**DOI:** 10.1186/s12906-023-03861-9

**Published:** 2023-01-28

**Authors:** Youngseok Ham, Tae-Jong Kim

**Affiliations:** Department of Forest Products and Biotechnology, College of Science and Technology, Kookmin University, 77 Jeongneung-ro, Seongbuk-gu, Seoul, 02707 Republic of Korea

**Keywords:** Biofilm, Glucosyltransferase, Glycyrrhizae Radix, Rubi Fructus, *Streptococcus mutans*

## Abstract

**Background:**

*Streptococcus mutans* is a bacterium that causes oral diseases. Plaque, a biofilm produced by *S. mutans* and other bacteria, makes it difficult to remove cariogenic oral microorganisms, including biofilm producers. Glucan synthesis by glucosyltransferase is one of the mechanisms underlying plaque formation. This study demonstrates the effectiveness of inhibiting biofilm formation by interfering with the glucosyltransferase activity of *S. mutans* using edible herbal medicines.

**Methods:**

This study investigated the inhibitory activity of Glycyrrhizae Radix extract, Rubi Fructus extract, glycyrrhizin from Glycyrrhizae Radix, and ellagic acid from Rubi Fructus against glucosyltransferase activity of *S. mutans*. Enzyme kinetic analysis identified the mechanism by which glycyrrhizin and ellagic acid inhibit enzyme activity.

**Results:**

The conditions for synergistically inhibiting biofilm formation by combining Glycyrrhizae Radix and Rubi Fructus extracts were identified. Biofilm formation was also synergistically inhibited by mixing their respective active constituents, glycyrrhizin and ellagic acid. Glycyrrhizin and ellagic acid inhibited glucosyltransferase via noncompetitive and uncompetitive mechanisms, respectively, indicating that they inhibit it via distinct mechanisms.

**Conclusions:**

This study presents an effective oral hygiene method using the synergistic activity of two natural plant extracts to inhibit biofilm formation through different inhibitory mechanisms against glucosyltransferase of *S. mutans*.

**Supplementary Information:**

The online version contains supplementary material available at 10.1186/s12906-023-03861-9.

## Background

Dental caries is an infectious oral disease that causes progressive destruction and loss of tooth enamel [1]. *Streptococcus mutans* is a major cause of dental caries and a representative bacterium that forms biofilm in the oral cavity, which is called plaque [2]. Furthermore, *S. mutans* glucosyltransferase (GTase) synthesizes sticky glucan from sucrose to promote the formation of dental biofilm and the attachment of oral microorganisms. Oral microorganisms, including *S. mutans*, produce organic acids from carbohydrates and accelerate the progression of dental caries [3]. Therefore, it is essential to inhibit the formation of dental biofilm to prevent or treat dental caries.

GTase synthesizes water-soluble and/or water-insoluble glucan. Exopolysaccharide-based insoluble glucan is a significant component in the formation of dental biofilm [4] and helps *S. mutans* adhere to the surface of teeth [5–7]. Because GTase uses sucrose to produce insoluble glucans, it has been suggested that sugar alcohols such as xylitol and maltitol [8, 9], alternative carbohydrates such as maltose and fructose [10, 11], and alternative sweeteners such as stevia [12] can substitute sucrose. GTase consists of two functional domains: the amino-terminal catalytic domain (CAT domain), which binds to and hydrolyzes sucrose, and the carboxyl-terminal glucan-binding domain, which binds to glucan and determines the properties of synthesized glucan [13, 14]. Inhibitors that prevent sucrose from binding to the CAT domain of GTase have been proposed to reduce plaque by inhibiting the production of insoluble glucan [15, 16].

In recent decades, chlorhexidine and sodium fluoride have been used to prevent oral diseases; however, they are irritative and have negative consequences, such as the emergence of resistant strains [17–19]. Alternatively, plant extracts have been proposed as novel materials for promoting oral health [20, 21] although they require high concentrations to achieve antibiotic-like efficacy. Due to their inherent flavor and aroma, consumers may reject high concentrations of plant extracts. To mitigate these issues without diminishing their efficacy, it is necessary to use plant extracts at lower concentrations. In this context, it has been proposed that extract combinations can be utilized [22–27]. However, few studies have examined combinations with the synergistic inhibition of *S. mutans* biofilm formation. In addition, it is necessary to identify the active constituents of plant extracts that can be used in combination and their biological mechanisms of action.

Our previous studies recently proposed two materials that strongly inhibit *S. mutans* biofilm formation via different mechanisms [28, 29]. One is a 50% ethanol extract of Glycyrrhizae Radix that inhibits the growth of *S. mutans* [29]. Its active constituents are glycyrrhizin and glycyrrhetic acid. The other is a methanol extract of Rubi Fructus (fruit of *Rubus coreanus*) that inhibits GTase activity but not the growth of *S. mutans* [28].

Glycyrrhizae Radix and Rubi Fructus are sweet, medicinal, or functional foods with a long history of use. In numerous studies, their biological activities and active constituents have also been suggested. Glycyrrhizae Radix is recognized for its anti-inflammatory [30], antioxidant [31], and hepatoprotective [32] properties, as well as its ability to prevent gastrointestinal ulcers [33] and alleviate dry mouth [34]. Meanwhile, Rubi Fructus has been reported to exert anti-inflammatory [35, 36], antioxidant [37, 38], and anticancer effects [39]. A lollipop containing Glycyrrhizae Radix extract has been developed for the prevention of caries [40]. Glycyrrhizae Radix is known to be particularly effective at preventing dental caries.

In this study, a method for inhibiting the biofilm formation of *S. mutans* by combining the extracts of Glycyrrhizae Radix and Rubi Fructus is proposed. To this end, this study proposes an extraction method for Rubi Fructus in addition to the extraction method for Glycyrrhizae Radix from the previous study [29], as well as a biological mechanism of component compounds that inhibit the biofilm formation of *S. mutans* in the two proposed extracts.

## Methods

### Strain, medium, and culture conditions

*Streptococcus mutans* GS-5, a serotype *c* strain, was provided by LG Household & Health Care Ltd. [41]. Brain heart infusion (BHI) medium (SKU: 237500) was purchased from Becton, Dickinson and Company Korea Ltd. (Seoul, Korea). BHI-S medium for biofilm formation was made using BHI medium and 1% (w/v) sucrose. BHI agar plates were made with BHI medium and 1.5% (w/v) agar. *S. mutans* stored in 25% (v/v) glycerol stock at − 80 °C was streaked on a BHI agar plate and incubated at 37 °C for 2 days. The BHI medium was inoculated with a single colony from the BHI agar plates and incubated at 37 °C for 24 h.

Glycorrhizin (catalog number: G0150) and ellagic acid dihydrate (catalog number: E0375) were purchased from Tokyo Chemical Industry Co., Ltd. (Tokyo, Japan) and used as standard compounds for the analyses of Glycyrrhizae Radix and Rubi Fructus, respectively. Glycyrrhizin (100 mM) and ellagic acid (5 mM) were dissolved in dimethyl sulfoxide (DMSO) before use.

### Preparation of Glycyrrhizae Radix and Rubi Fructus extracts

Dried Glycyrrhizae Radix, purchased from Jiwoondang Herbal Medicine Store (Seoul, Korea), was ground to a powder of ≤1 mm. Then, the extract solution was mixed at 8 mL per 1 g of Glycyrrhizae Radix powder and incubated at 83 °C for 3 h. The solid powder was eliminated by filtration using a 75 μm–pore cartridge filter. The solvent was mixed again at 2 mL per 1 g of Glycyrrhizae Radix powder and incubated at 83 °C for 2 h. The residual powder was removed once more with the use of the same cartridge filter. The powdered mixture of the filtered primary and secondary extracts was produced via spray drying.

Dried Rubi Fructus from Jiwoondang Herbal Medicine Store (Seoul, Korea) was ground to a powder with a particle size of ≤1 mm. Next, the solvent was mixed at 10 mL per 1 g of the Rubi Fructus powder and incubated at 100 °C (water) or 70 °C (50 and 90% ethanol) for 3 h. Following filtration with a cartridge filter, the extract was spray-dried into a powder. The solid yields were 12.7% with water, 21.2% with 50% ethanol, and 10.7% with 90% ethanol.

Glycyrrhizae Radix and Rubi Fructus were formally identified by the Herbal Medicine Store, where they were purchased. All extract powders were vacuum-sealed and stored at − 80 °C until use. Before use, extract powders of Glycyrrhizae Radix and Rubi Fructus were dissolved in distilled water.

### Biofilm formation and its quantitative analysis

Microplate biofilm formation was measured using crystal violet staining method [42]. In order to create biofilm, 100 μL of BHI-S medium was dispensed into each well of a 96-well polyvinylchloride microplate, and 5% of the sample was added. *S. mutans*, with an absorbance of 0.05 at 600 nm, was inoculated into each well. After 24 h of incubation at 37 °C, the absorbance at 595 nm was measured to determine cell growth using an Opsys MR microplate reader (DYNEX Technologies, Chantilly, VA, USA). The planktonic cells were then washed with distilled water. The biofilm was stained with 1% crystal violet and then rinsed with distilled water. For the quantitative analysis of biofilm formation, crystal violet remaining in the biofilm was eluted with 95% ethanol, and the absorbance at 595 nm was measured. The relative amount of biofilm was calculated by comparison with the control value.

### Measuring glucosyltransferase activity

Glucosyltransferase activity was measured using a modified version of the method described in previous reports [43, 44]. *S. mutans* which was stored at − 80 °C, was inoculated on a BHI agar plate and cultured at 37 °C for 2 days. A single colony was inoculated into 40 mL of BHI medium and incubated at 37 °C for 24 h. The preculture cells were inoculated onto 1 L of BHI medium to reach Abs_600_ = 0.05 and incubated at 37 °C for 24 h. The cells were separated from the supernatant using a centrifuge (Combi-514R; Hanil Science Industrial Co., Ltd., Daejeon, Korea) at 1500×g and 4 °C for 10 min. This cell-free supernatant was combined with cold 95% ethanol and incubated at 4 °C for 24 h. The supernatant was discarded after centrifugation at 1500×g and 4 °C for 10 min to obtain precipitated GTase. Precipitated GTase was washed with 10 mL of 60 mM potassium phosphate buffer (pH 6.8). The GTase was homogenized with an additional 10 mL of 60 mM potassium phosphate buffer (pH 6.8) and stored at − 80 °C.

By analyzing the amount of insoluble glucan produced from sucrose during the enzymatic reaction, the GTase activity was determined. The substrate for the reaction was 1.25% (w/v) sucrose in a 60 mM potassium phosphate buffer at pH 6.8 with 0.025% NaN_3_. For the reaction, 800 μL of the substrate solution, 50 μL of GTase, 50 μL of the testing sample, and 100 μL of distilled water were mixed and incubated at 37 °C for 24 h. After incubation, insoluble glucan was homogenized for 5 s using a Sonic Dismembrator (model 100; Thermo Fisher Scientific Inc., Waltham, MA, USA) with the power of scale 4. After centrifugation at 1500×g and 4 °C for 10 min, most of the supernatant was removed, leaving ~ 200 μL. The pelleted insoluble glucan was homogenized using sonication under the same conditions mentioned above. After transferring 200 μL into a 96-well polystyrene microplate (catalog number: CLS3628; Corning Inc., New York, NY, USA), absorbance at 540 nm was measured using an Opsys MR™ microplate reader. For the control experiments, distilled water was substituted for the test sample. To calculate the relative GTase activity, the sample value was compared to the control value.

### Content analysis of ellagic acid by high-performance liquid chromatography

The concentration of ellagic acid in the Rubi Fructus extract was determined using high-performance liquid chromatography (HPLC) in accordance with the method described in a previous report [45]. Each specimen was filtered using a SEPARA® vial filter (catalog number: MV32ANPPV002FC01; GVS Korea Ltd., Namyangju, Korea). The analytical column was YMC-Triart C18 (catalog number: TA12S05-2546WT; YMC Korea Co., Ltd., Seongnam, Korea). The HPLC instrument was Acme 9000 HPLC, which comprises a vacuum degasser and mixer (catalog number: SDV40A), gradient pump oven (catalog number: CTS30), auto-sampler [catalog number: YL9150 AS (Alias)], solvent delivery pump (catalog number: SP930D), and UV/Vis detector with dual wavelength (catalog number: YL9120) from Young Lin Instrument Co. (Anyang, Korea). The mobile solvents were 1% acetic acid (A) and acetonitrile (B). The following gradient steps were utilized for chemical separation: 90% (A):10% (B) at 0 min; 60% (A):40% (B) at 28 min; 40% (A):60% (B) at 39 min; 10% (A):90% (B) at 50 min; and 90% (A):10% (B) at 55 min. The flow rate of the mobile phase was 0.7 mL/min, and a UV absorbance detector was used to detect the chemicals at 272 nm. The amount of sample injected was 10 μL, and the temperature of the analytical column was 30 °C. The data were analyzed using Autochro-3000 software version 2.0.0 (Young Lin Instrument Co.).

### Evaluating synergistic biofilm formation inhibition using a checkerboard microdilution assay

Using a 96-well microplate, the synergistic activity of extract combinations was evaluated [46]. The relative decrease in biofilm formation due to a sample was calculated by comparison with the amount of biofilm formation of the untreated control [47]. Synergistic activity was determined to occur when the relative inhibition value of a combination was more than double the relative inhibition value of each sample.

### Kinetic analysis of GTase activity

Michaelis–Menten constants (*K*_m_) and maximum reaction rate (*V*_max_) of GTase were determined using a slightly modified version of a previously described method [48]. At 37 °C for 30 min, GTase activity was measured with sucrose concentrations ranging from 0 to 1%, and the values of *V*_max_ and *K*_m_ were calculated using the Lineweaver–Burk equation. To determine the type of inhibition, the rate of change of the reaction upon the addition of glycyrrhizin or ellagic acid was compared.

### Statistical analyses

To calculate the mean value and standard deviation for all experimental results, the statistical program IBM® SPSS software (Ver. 25.0; SPSS Inc., Chicago, IL, USA) was utilized (SPSS Inc., Chicago, IL, USA). The results were determined using the *t*-test at the 95% confidence level.

## Results and discussion

### Inhibitory activity of Rubi Fructus extracts on biofilm formation and glucosyltransferase of *S. mutans*

Methods for preventing oral diseases caused by *S. mutans* include 1) inhibiting the growth of *S. mutans*, 2) inhibiting the biofilm formation of *S. mutans* (including inhibiting glucan synthesis by GTase), and 3) employing alternative sweeteners that cannot be exploited by *S. mutans* [49].

In a previous study, ellagic acid rhamnoside from *R. ulmifolius* was found to inhibit the biofilm formation of *Staphylococcus aureus* [50], and the *R. idaeus* extract inhibited the biofilm formation of *Candida albicans* without antifungal activity [51]. However, Rubi Fructus extract did not inhibit the biofilm formation of seven bacterial strains: *Pseudomonas libanensis*, *Serratia marcescens*, *Acinetobacter* sp., *Bacillus amyloliquefaciens*, *Hafnia paralvei*, *Obesumbacterium proteus*, and *Pseudomonas aeruginosa* [52]. These findings suggest that the inhibitory activity of *Rubus* spp. extract against microbial biofilm is strain-specific.

**Fig. 1:**
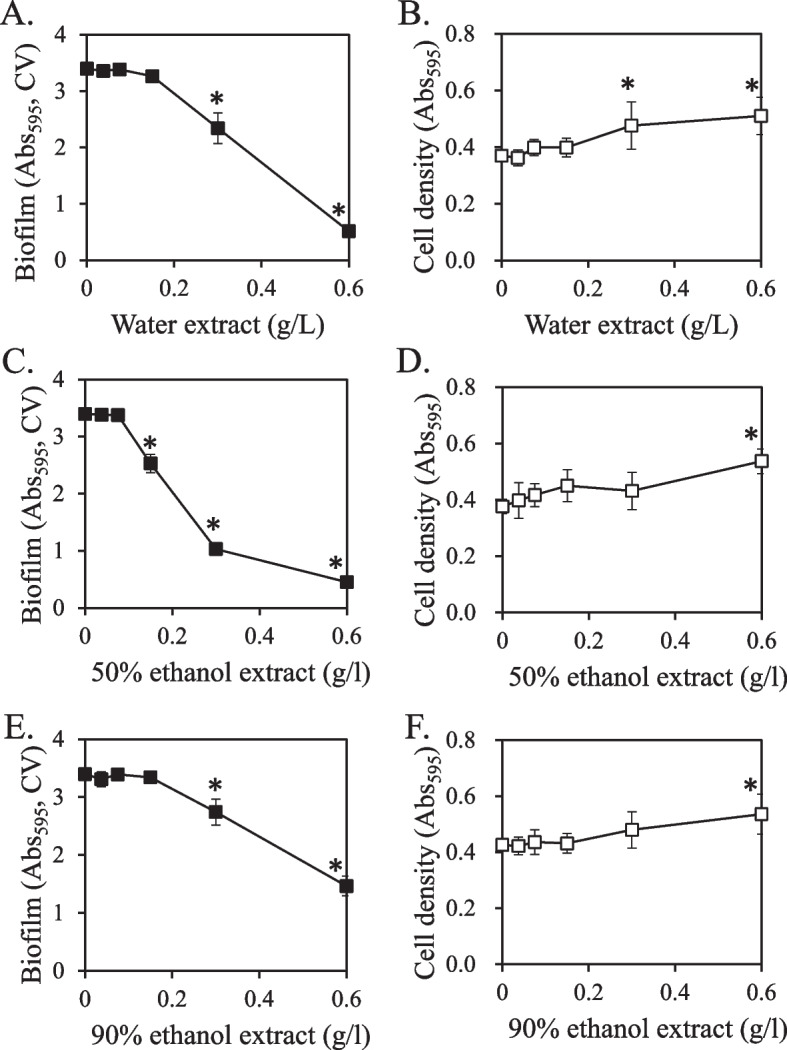
Effect of Rubi Fructus extracts on biofilm formation and *S. mutans* growth. The quantity of biofilm was represented by crystal violet (CV) absorbance at 595 nm. Values that differ from the control with a 95% confidence level are denoted with an asterisk on top of the symbols. **A** biofilm production with water extract; **B** cell growth with water extract; **C** biofilm production with 50% ethanol extract; **D** cell growth with 50% ethanol extract: **E** biofilm formation with 90% ethanol extract; **F** cell growth with 90% ethanol extract

**Fig. 2:**
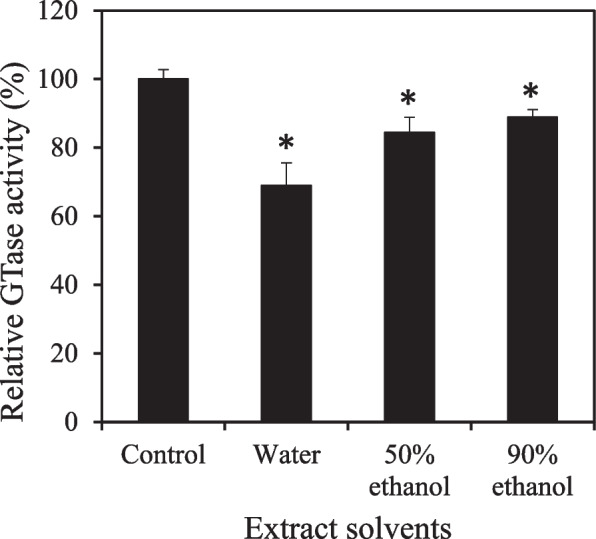
Effect of Rubi Fructus extract on the GTase activity of *S. mutans.* GTase activity was determined by measuring the amount of insoluble glucan at a wavelength of 540 nm. The tested concentration of all extract was 2.5 g/L. Values that differ from the control with a 95% confidence level are marked with an asterisk on top of the bars

Our previous study found that the methanol extract of Rubi Fructus reduced the GTase activity and biofilm formation of *S. mutans* [28]. However, methanol is an unsuitable solvent for the production of edible products. This study prepared a Rubi Fructus extract using water and ethanol as extraction solvents. *S. mutans* biofilm formation (Fig. 1) and GTase activity (Fig. 2) were found to be effectively inhibited by a water extract of Rubi Fructus. Biofilm formation was inhibited in a concentration-dependent manner by all Rubi Fructus extracts, but the growth of *S. mutans* was not inhibited at any of the concentrations examined (Fig. 1).

### Ellagic acid of Rubi Fructus inhibiting biofilm formation and GTase activity of *S. mutans*

In previous studies, ellagic acid was shown to inhibit the biofilm formation of *Escherichia coli* [53], *S. aureus* [54], and *Cutibacterium acnes* [55]. In this study, the ellagic acid content of the extract was analyzed based on the solvent used for extraction (Table 1). The ellagic acid concentration in the water extract was the highest at 1.55 mmol/g, whereas it decreased as the ethanol concentration in the extracted solvent increased. The GTase inhibitory activity of Rubi Fructus extract was proportional to its ellagic acid content (Table 1 and Fig. 2).

**Table. 1:** HPLC analysis of ellagic acid content of Rubi Fructus extract

Extract solvent	Water	50% ethanol	90% ethanol
Extraction yield (%)^a)^	12.7	21.2	10.7
Ellagic acid concentration (mmol/g)^b)^	1.55	1.17	1.10

^a)^ Extraction yield (%) = weight of solid after extraction (g) / weight of Rubi Fructus used (g) × 100

^b)^ Amount of ellagic acid per gram of Rubi Fructus extract

**Fig. 3:**
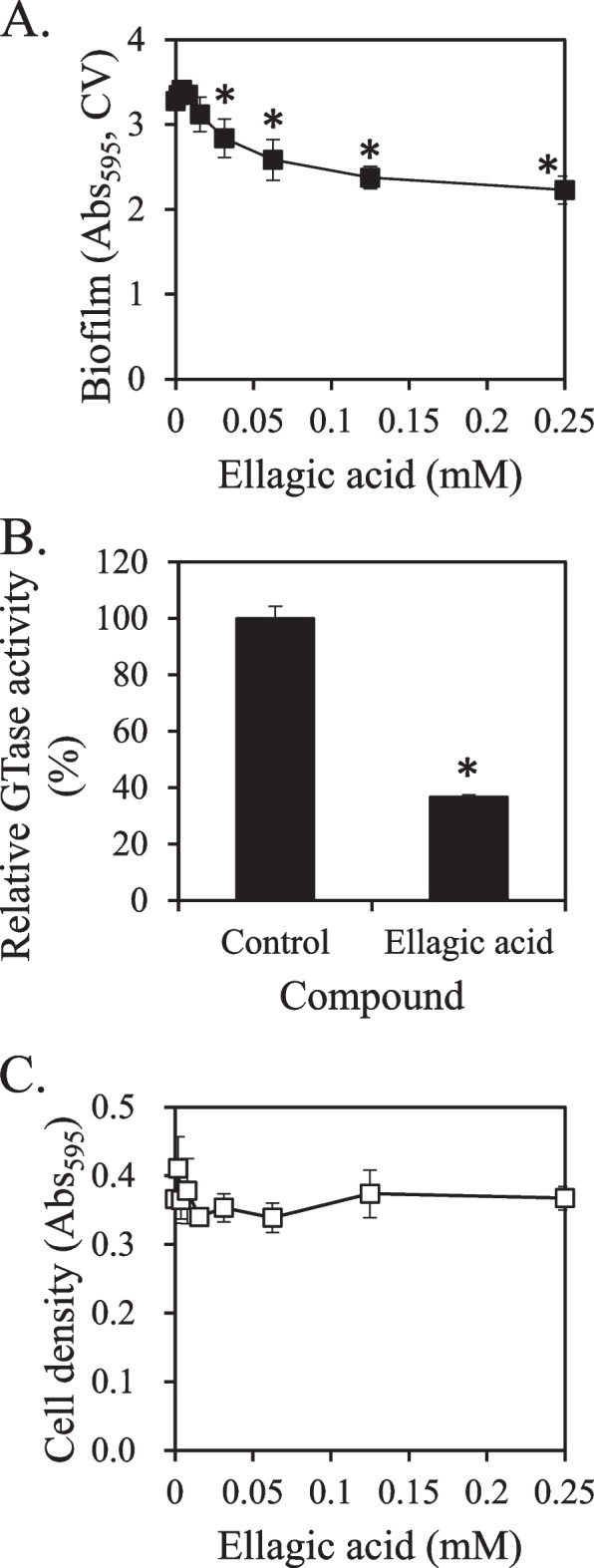
Effect of ellagic acid on *S. mutans* biofilm formation (**A**), cell growth (**B**), and GTase activity (**C**). At a concentration of 0.25 mM, the impact of ellagic acid on GTase activity was examined (**B**). The amount of biofilm was represented by crystal violet (CV) absorbance at 595 nm. Six (**A** and **C**) and three (**B**) distinct experiments were used to calculate the values. **A** and **B** were analyzed statistically using Tukey’s test (**A** and **B**) or a paired t-test (**C**). Values that differ from the control with a 95% confidence level are marked with an asterisk on top of the symbols or bars

The 0.25-mM elagic acid concentration decreased biofilm formation by 32% (Fig. 3A) and GTase activity by 63%. (Fig. 3B). However, ellagic acid did not inhibit the growth of *S. mutans* in the same way that Rubi Fructus extract did (Fig. 3C). According to a previous study, ellagic acid inhibits the GTase activity of *S. mutans* [56] and *S. sobrinus* [57]. This study demonstrates that water is a superior extraction solvent for Rubi Fructus to inhibit biofilm formation and GTase activity of *S. mutans* and that ellagic acid is the active compound responsible for the extract’s inhibitory activity.

Rubi Fructus extract did not inhibit the growth of *S. mutans* (Fig. 1), nor did ellagic acid up to 0.25 mM (0.076 mg/mL) affect the growth (Fig. 3C). However, it has been reported that the minimum inhibitory concentration (MIC) of ellagic acid against oral bacteria, *S. mutans*, *S. sanguinis*, and *S. salivarius* is < 1 mg/mL, making it more sensitive than *Actinomyces viscosus* and *Lactobacillus rhamnosus* [58]. Notably, 30-μg/mL ellagic acid had no effect on the growth of *E. coli* but it inhibited biofilm formation [53]. Low MIC concentrations and biofilm inhibition for ellagic acid in a previous study suggest that ellagic acid can specifically inhibit the growth of cariogenic oral streptococci, such as *S. mutans*, *S. sanguinis*, and *S. salivarius*.

### Synergistic inhibitory activity of Rubi Fructus and Glycyrrhizae Radix extracts

**Fig. 4:**
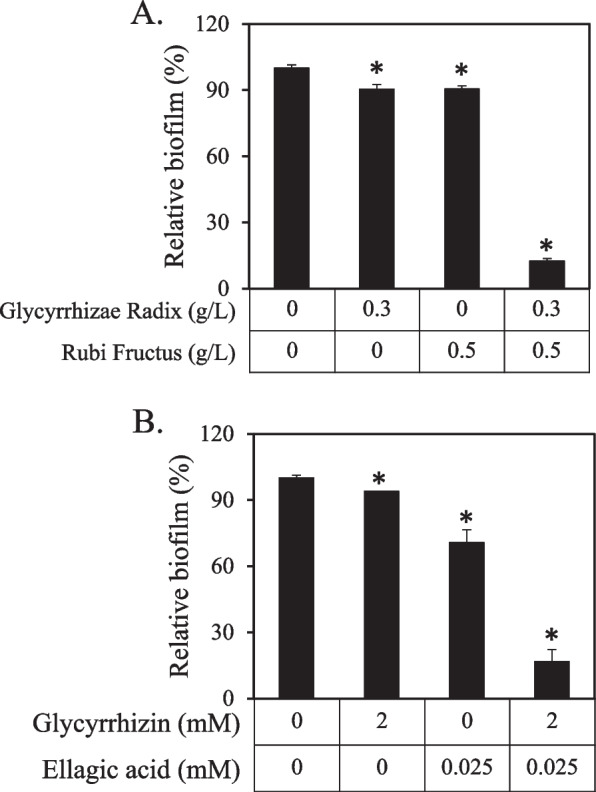
Synergistic inhibition of biofilm formation by extract combinations against *S. mutans*. The synergistic inhibitory activity of Glycyrrhizae Radix extract and Rubi Fructus extract (**A**) and glycyrrhizin and ellagic acid (**B**) against the formation of biofilm by *S. mutans*. A control was administered, either distilled water (**A**) or DMSO (**B**). The amount of biofilm was measured by the absorbance of crystal violet (CV) at 595 nm. Values that differ from the control with a 95% confidence level are marked with an asterisk on top of the bars

A 50% ethanol extract of Glycyrrhizae Radix inhibited the growth and biofilm formation of *S. mutans* in a previous study [29] but had no effect on GTase activity (Supplementary Fig. 1). The extract yield of Glycyrrhizae Radix used was 19% in terms of solid content. These findings suggested that Glycyrrhizae Radix extract and Rubi Fructus extract exert different inhibitory effects on the biofilm formation and GTase activity of *S. mutans* via distinct mechanisms, suggesting that they can be combined for synergistically increased effectiveness. The optimal combination ratio synergistically inhibiting biofilm formation was identified using the checkerboard method (Supplementary Table 1). The combination of Glycyrrhizae Radix extract at 0.3 g/L and Rubi Fructus extract at 0.5 g/L exhibited synergistic activity (Fig. 4A). The activity of this combination was 9.1 times more effective than Glycyrrhizae Radix extract alone and 9.2 times more effective than Rubi Fructus extract alone.

Glycyrrhizin of Glycyrrhizae Radix has been reported to inhibit the cell growth, biofilm formation, and GTase activity of *S. mutans* [29, 59]. The combination of glycyrrhizin and ellagic acid with synergistic inhibition of biofilm formation was also identified using the checkerboard technique (Supplementary Table 2). The activity of inhibiting biofilm formation was increased 13.7-fold and 2.8-fold, respectively, when 2 mM glycyrrhizin and 0.025 mM ellagic acid were combined, compared to the activity of each compound alone (Fig. 4B). Considering the synergistic activity of glycyrrhizin and ellagic acid on *S. mutans*, they may contribute to the synergistic activity of Glycyrrhizae Radix and Rubi Fructus. MIC for Glycyrrhizae Radix extract against cavity-causing bacteria such as *S. mutans*, *S. sobrinus*, and *Lactobacillus casei* [40] was between 15.6 and 31.2 μg/mL. Lollipops containing Glycyrrhizae Radix extract decreased cavity-causing bacteria in the oral cavity [40]. In addition, Glycyrrhizae Radix extract inhibited the growth of *B. subtilis* and *C. acnes* [60], indicating that it selectively inhibited the growth of bacteria and was not limited to oral microorganisms. Glycyrrhizin, a component of Glycyrrhizae Radix, has been demonstrated to have bactericidal activity against *E. coli* [61], *B. subtilis* [62], and *S. epidermidis* [62]. β-Glycyrrhetinic acid had MICs ranging from 16 to 512 μg/mL for cariogenic oral streptococci, such as *S. mutans*, *S. sobrinus*, *S. anginosus*, *S. sanguinis*, *S. salivarius*, *S. gordonii*, and *S. oralis* [63].

### Enzymatic inhibitory mechanism of glycyrrhizin and ellagic acid on GTase

**Fig. 5:**
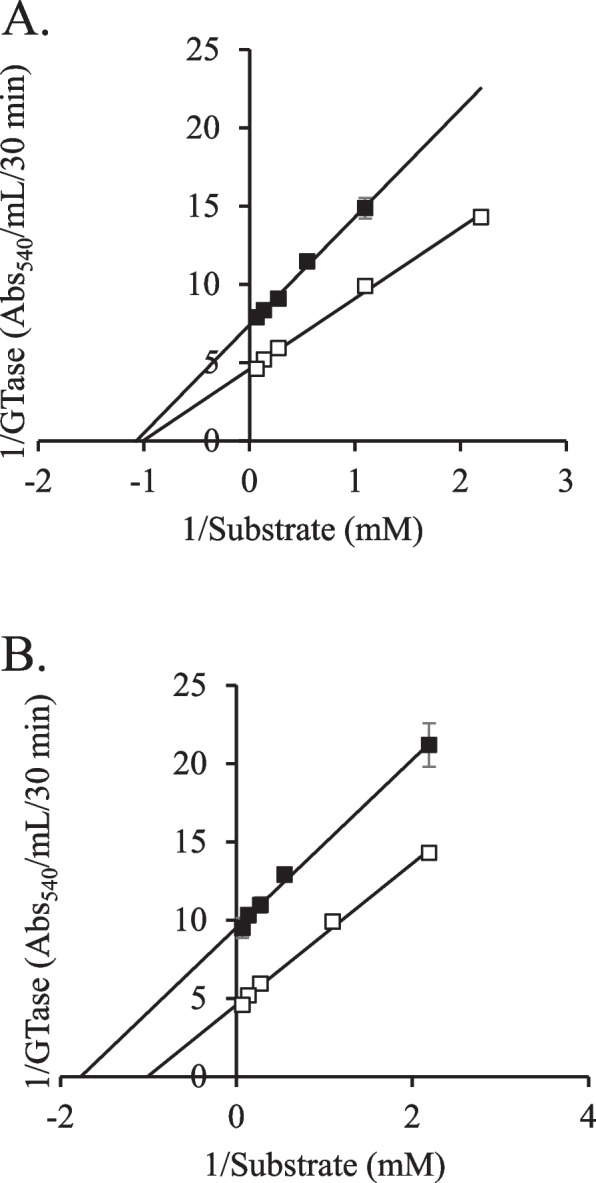
Inhibitory effects of glycyrrhizin (**A**) and ellagic acid (**B**) on the kinetics of the enzyme GTase. Changes in GTase activity were evaluated without (open square) and with chemicals (filled square). The y-axis represents the inverse of GTase activity, while the x-axis represents the inverse of substrate concentration (mM). GTase activity is measured as the quantity of insoluble glucan produced in 1 mL over a period of 24 h. The Lineweaver–Burk equations for control, glycyrrhizin, and ellagic acid were 1/*V* = 4.52 × 1/[*S*] + 4.58, 1/*V* = 6.90 × 1/[*S*] + 7.40, and 1/*V* = 5.38 × 1/[*S*] + 9.51, respectively. The values were calculated from three independent experiments.

To determine the Michaelis–Menten kinetic constant (*K*_m_) and maximum velocity (*V*_max_) of GTase isolated from *S. mutans*, the enzymatic activity was measured using various concentrations of sucrose, and the Lineweaver–Burk equation was used to plot the obtained results (Fig. 5). Without any control compounds, the *K*_m_ and *V*_max_ values of GTase were 0.99 mM and 0.218 Abs_540_/mL/30 min, respectively. The *K*_m_ value for glycyrrhizin was 0.93 mM, which was comparable to that of the control GTase. However, the *K*_m_ value for ellagic acid was 0.57 mM, which was 42% lower than that of the control GTase. *V*_max_ values for glycyrrhizin and ellagic acid were 0135 and 0.105 Abs_540_/mL/30 min, representing decreases of 38 and 52%, respectively. These results indicated that glycyrrhizin is a noncompetitive inhibitor and ellagic acid is an uncompetitive inhibitor of GTase, indicating that these two compounds inhibit the enzyme in different ways.

In previous studies, inhibitors of GTase activity with diverse inhibitory mechanisms were described. For instance, polyphenols of oolong tea noncompetitively inhibited the glucan-binding domain of the GTase of *S. mutans* [64]. Meanwhile, tannic acid, gallic acid, and syringic acid inhibited the GTase activity of *S. mutans* through mixed-type inhibition [48]. Furthermore, 7-epiclusianone inhibited GTase B activity by mixed-type inhibition while noncompetitively inhibiting GTase C activity [65]. According to our previous research, vanillic acid, ferulic acid, and salicylic acid were uncompetitive, competitive, and noncompetitive inhibitors, respectively [28]. In this experiment, the addition of glycyrrhizin and ellagic acid from Glycyrrhizae Radix and Rubi Fructus did not significantly change pH. This study presents two different inhibitors of GTase, glycyrrhizin, a noncompetitive inhibitor, and ellagic acid, a noncompetitive inhibitor. *S. mutans* biofilm formation was inhibited synergistically by extracts of Glycyrrhizae Radix and Rubi Fructus containing these two different inhibitors as the main active substances, respectively.

## Conclusions

This study revealed the synergistic activity of a combination of two different natural compounds as a potential method for inhibiting *S. mutans’* formation of dental biofilm. Rubi Fructus water extract inhibited GTase activity and biofilm formation without inhibiting the growth of *S. mutans*. Glycyrrhizin and ellagic acid were found to be noncompetitive and uncompetitive inhibitors of *S. mutans* GTase, respectively. The combination of glycyrrhizin of Glycyrrhizae Radix and ellagic acid of Rubi Fructus synergistically inhibited biofilm formation, and the combination of Rubi Fructus extract with Glycyrrhizae Radix extract exerting different mechanisms of action showed a strong ability to inhibit biofilm formation at low concentrations. Thus, it is possible to develop effective products for preventing oral diseases caused by *S. mutans* dental biofilm using the proposed combination’s synergistic activity.

## Supplementary Information

**Additional file 1: Supplementary Fig. 1.** Effect of Glycyrrhizae Radix extract at 2.5 g/L on GTase activity of *S. mutans*.

**Additional file 2: Supplementary Table 1.** Synergistic inhibitory activity of extract combination, Glycyrrhizae Radix extract and Rubi Fructus extract, against the biofilm formation of *S. mutans* with checkerboard assay. This table shows the results for all tested concentration combination of Glycyrrhizae Radix extract and Rubi Fructus extract. After quantitatively analyzing the amount of biofilm with crystal violet, the relative value was calculated by comparing it with the control value. The control value was the amount of biofilm produced without any treatment. The values were calculated from three independent experiments. The shaded cell is a concentration condition that synergistically inhibited biofilm formation. **Supplementary Table 2.** Synergistic inhibitory activity of compound combination, glycyrrhizin and ellagic acid, against the biofilm formation of *S. mutans* with checkerboard assay. This table shows the results for all tested concentration combination of glycyrrhizin and ellagic acid. After quantitatively analyzing the amount of biofilm with crystal violet, the relative value was calculated by comparing it with the control value. The control value was the amount of biofilm produced without any treatment. The values were calculated from three independent experiments. Shaded cells are concentration conditions that synergistically inhibited biofilm formation.

## Data Availability

The datasets used and/or analyzed during the current study are available from the corresponding author upon reasonable request.

## Abbreviations

BHI: Brain Heart Infusion
CAT domain: Catalytic domain
DMSO: Dimethyl sulfoxide
GTase: Glucosyltransferase
HPLC: High-performance liquid chromatography
*K*_m_: Michaelis–Menten constants
*V*_max_: Maximum reaction rate
