# Supplementary material for: Synergistic inhibitory activity of Glycyrrhizae Radix and Rubi Fructus extracts on biofilm formation of Streptococcus mutans
Source: BMC Complement Med Ther. 2023 Jan 28;23:22. doi: 10.1186/s12906-023-03861-9 (PMC9883881; doi:10.1186/s12906-023-03861-9)
Supplement: Supplementary file 1 — Additional file 1: Supplementary Fig. 1. Effect of Glycyrrhizae Radix extract at 2.5 g/L on GTase activity of S. mutans. [file 12906_2023_3861_MOESM1_ESM.pptx]

## Slide 1
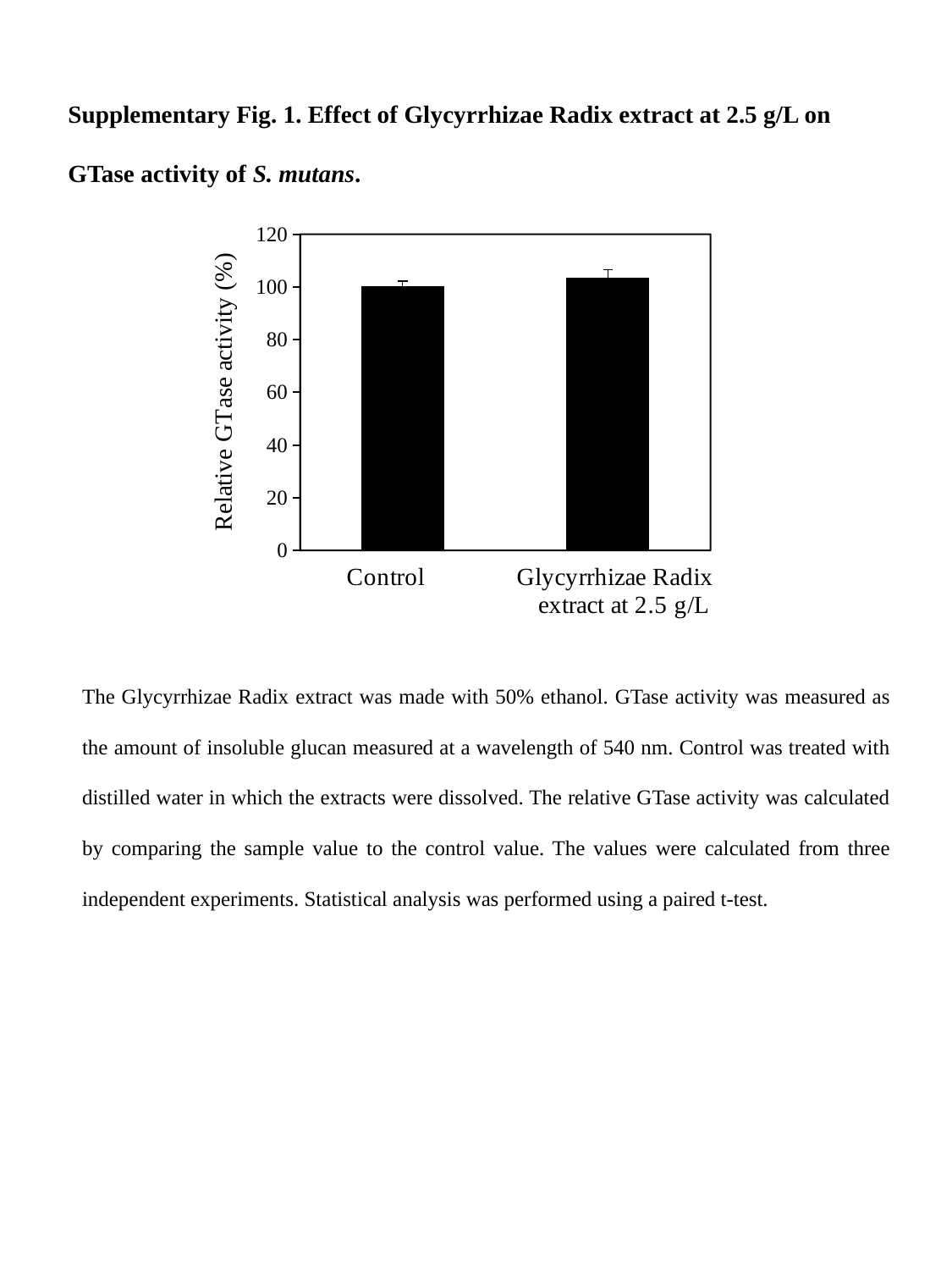

Supplementary Fig. 1. Effect of Glycyrrhizae Radix extract at 2.5 g/L on GTase activity of S. mutans.
### Chart
| Category | Licorice |
|---|---|
| Control | 100.00000000000001 |
| Licorice 50% ethanol | 103.16027088036121 |The Glycyrrhizae Radix extract was made with 50% ethanol. GTase activity was measured as the amount of insoluble glucan measured at a wavelength of 540 nm. Control was treated with distilled water in which the extracts were dissolved. The relative GTase activity was calculated by comparing the sample value to the control value. The values were calculated from three independent experiments. Statistical analysis was performed using a paired t-test.
