# Supplementary material for: Synergistic inhibitory activity of Glycyrrhizae Radix and Rubi Fructus extracts on biofilm formation of Streptococcus mutans
Source: BMC Complement Med Ther. 2023 Jan 28;23:22. doi: 10.1186/s12906-023-03861-9 (PMC9883881; doi:10.1186/s12906-023-03861-9)
Supplement: Supplementary file 2 — Additional file 2: Supplementary Table 1. Synergistic inhibitory activity of extract combination, Glycyrrhizae Radix extract and Rubi Fructus extract, against the biofilm formation of S. mutans with checkerboard assay. This table shows the results for all tested concentration combination of Glycyrrhizae Radix extract and Rubi Fructus extract. After quantitatively analyzing the amount of biofilm with crystal violet, the relative value was calculated by comparing it with the control value. The control value was the amount of biofilm produced without any treatment. The values were calculated from three independent experiments. The shaded cell is a concentration condition that synergistically inhibited biofilm formation. Supplementary Table 2. Synergistic inhibitory activity of compound combination, glycyrrhizin and ellagic acid, against the biofilm formation of S. mutans with checkerboard assay. This table shows the results for all tested concentration combination of glycyrrhizin and ellagic acid. After quantitatively analyzing the amount of biofilm with crystal violet, the relative value was calculated by comparing it with the control value. The control value was the amount of biofilm produced without any treatment. The values were calculated from three independent experiments. Shaded cells are concentration conditions that synergistically inhibited biofilm formation. [file 12906_2023_3861_MOESM2_ESM.docx]

**Supplementary Table 1.** Synergistic inhibitory activity of extract combination, Glycyrrhizae Radix extract and Rubi Fructus extract, against the biofilm formation of *S. mutans* with checkerboard assay. This table shows the results for all tested concentration combination of Glycyrrhizae Radix extract and Rubi Fructus extract. After quantitatively analyzing the amount of biofilm with crystal violet, the relative value was calculated by comparing it with the control value. The control value was the amount of biofilm produced without any treatment. The values were calculated from three independent experiments. The shaded cell is a concentration condition that synergistically inhibited biofilm formation.

|  | Glycyrrhizae Radix (g/L) | 0.5 | 0.4 | 0.3 | 0.2 | 0.1 | 0.05 |
| --- | --- | --- | --- | --- | --- | --- | --- |
| Rubi Fructus (g/L) | Biofilm inhibition (%) | 1.1 ± 1.2 | 24.1 ± 1.3 | 90.4 ± 1.0 | 99.8 ± 0.8 | 99.8 ± 0.7 | 99.2 ± 0.0 |
| 1 | 17.6 ± 1.3 | 0.4 ± 0.1 | 0.1 ± 0.5 | 0.3 ± 0.1 | 9.9 ± 1.7 | 15.8 ± 2.6 | 17.6 ± 2.0 |
| 0.9 | 19.2 ± 2.5 | 0.5 ± 0.2 | 0.4 ± 0.2 | 0.3 ± 0.1 | 8.5 ± 4.8 | 15.7 ± 1.5 | 18.0 ± 0.4 |
| 0.8 | 16.8 ± 3.5 | 0.8 ± 0.2 | 0.0 ± 2.7 | 0.6 ± 0.5 | 10.4 ± 2.3 | 14.6 ± 2.1 | 19.4 ± 2.4 |
| 0.7 | 23.4 ± 2.5 | 0.5 ± 0.3 | 0.3 ± 0.4 | 2.3 ± 3.1 | 24.5± 0.2 | 24.8 ± 3.6 | 39.8 ± 3.1 |
| 0.6 | 49.0 ± 2.0 | 0.5 ± 0.5 | 4.0 ± 5.7 | 5.4 ± 7.2 | 82.1 ± 2.1 | 81.5 ± 3.3 | 89.9 ± 3.6 |
| 0.5 | 90.5 ± 2.2 | 0.0 ± 0.2 | 0.1 ± 0.4 | 12.5 ± 2.9 | 96.1 ± 0.9 | 99.6 ±0.1 | 99.2 ± 0.4 |

**Supplementary Table 2.** Synergistic inhibitory activity of compound combination, glycyrrhizin and ellagic acid, against the biofilm formation of *S. mutans* with checkerboard assay. This table shows the results for all tested concentration combination of glycyrrhizin and ellagic acid. After quantitatively analyzing the amount of biofilm with crystal violet, the relative value was calculated by comparing it with the control value. The control value was the amount of biofilm produced without any treatment. The values were calculated from three independent experiments. Shaded cells are concentration conditions that synergistically inhibited biofilm formation.

|  | Glycyrrhizin (mM) | 5 | 4 | 3 | 2 | 1 | 0.5 |
| --- | --- | --- | --- | --- | --- | --- | --- |
| Ellagic acid  (mM) | Biofilm inhibition (%) | 14.8 ± 1.2 | 12.7 ± 1.9 | 45.3 ± 4.7 | 93.9 ± 0.0 | 98.4 ± 3.6 | 96.4 ± 3.7 |
| 0.25 | 46.6 ± 4.1 | 4.1 ± 1.4 | 3.7 ± 1.8 | 10.8 ± 4.1 | 11.3 ± 1.9 | 31.5 ± 0.4 | 38.2 ± 4.0 |
| 0.2 | 55.4 ± 4.6 | 2.7 ± 1.5 | 2.2 ± 1.1 | 12.3 ± 1.9 | 17.7 ± 7.5 | 29.5 ± 0.5 | 38.5 ± 1.8 |
| 0.15 | 58.5 ± 4.0 | 3.5 ± 1.4 | 2.5 ± 1.5 | 14.8 ± 0.3 | 15.3 ± 5.1 | 31.4 ± 2.2 | 46.2 ± 4.6 |
| 0.1 | 60.3 ± 3.6 | 5.0 ± 3.2 | 3.6 ± 0.2 | 12.7 ± 0.8 | 19.8 ± 9.8 | 34.1 ± 5.1 | 50.0 ± 7.6 |
| 0.05 | 68.7 ± 5.3 | 6.2 ± 3.5 | 2.8 ± 1.2 | 13.2 ± 1.9 | 26.3 ± 8.0 | 36.8 ± 0.3 | 57.4 ± 3.0 |
| 0.025 | 70.7 ± 5.8 | 4.2 ± 3.1 | 3.7 ± 1.8 | 10.1 ± 0.3 | 18.2 ± 7.6 | 22.5 ± 11.5 | 39.6 ± 7.6 |
